# A Case and Review of Noma

**DOI:** 10.1371/journal.pntd.0000869

**Published:** 2010-12-21

**Authors:** Joseph E. Tonna, Matthew R. Lewin, Brett Mensh

**Affiliations:** 1 Stanford/Kaiser Emergency Medicine Residency, Stanford University, Stanford, California, United States of America; 2 Institute for Exploration and Travel Health, California Academy of Sciences, San Francisco, California, United States of America; Emory University, United States of America

**Key Learning Points**

- Noma, or cancrum oris, is a rapidly progressive bacterial disease that results in painless gangrenous necrosis of the mouth.
- Noma is a disease of multifactorial etiology that presupposes a state of malnutrition; historically, it has been found worldwide during times of poverty, starvation, and war, including within the concentration camps of WWII.
- Estimated incidence of noma is 100,000 to 140,000 per year with a worldwide prevalence documented at over 700,000 cases. This may severely underestimate the actual figures, though, as it is believed that fewer than 15% of acute cases present for care.
- Untreated, acute noma in children is 80%–90% lethal. Antibiotic treatment and nutritional support decreases mortality to less than 10%. Nevertheless, plastic orofacial reconstruction is often necessary for both functionality and cosmesis.

## Case Description

**Figure 1:**
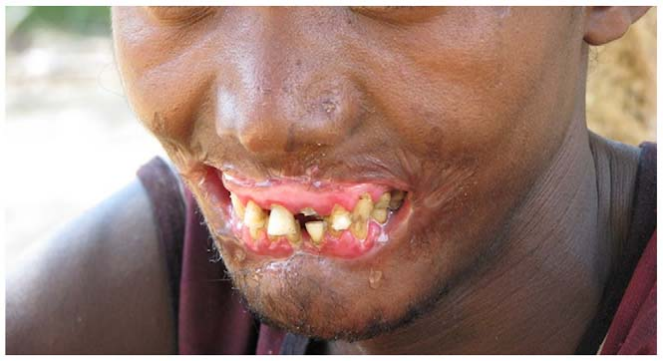
Young man with destructive perioral lesion and oral incontinence. Photo courtesy of Brian L. Fisher, California Academy of Sciences.

A young adult male villager in Madagascar developed painful, perioral swelling over a period of weeks. The stomatitis subsequently evolved into an anesthetic, rapidly expansive, necrotic ulcer with oral incontinence (Figure 1). A period of social instability during the preceding months likely caused malnutrition that resulted in significant weight loss.

## Diagnosis

Noma.

## Discussion

### Introduction

Noma, or cancrum oris, is a multi-stage,opportunistic, rapidly progressive, and painless necrotizing stomatitis. There are isolated reports of noma affecting other areas of the body, including the perineum, vulva, scalp, neck, and shoulders [1], [2].

### History and Epidemiology

Historically, noma is a disease of extreme poverty and malnourishment, reported throughout history in Asia, Europe, South America, and Africa [1], [3], [4]. It was described by Hippocrates [5] and found in German and Japanese concentration camps during World War II [1], [6]. Infection occurs mostly in children, although it has been described in neonates, adults, and the chronically ill [3], [7].

The true prevalence and incidence of noma are not fully known, because it is believed that only <15% of patients with acute cases of the disease seek medical care [1], [8]. Noma is a disease of shame, and the condition often results in forced isolation from the community and family; many children are sent to live in isolation rather than being taken to medical care [1], [8]–[10]. In the late 1990s, the incidence of acute childhood noma was placed at 25,600 in countries bordering the Sahara [11], and worldwide at between 100,000 and 140,000 per year, primarily in sub-Saharan Africa and Asia [12]. Peak incidence is among children aged 1–4 [3], [4], [7]. Worldwide prevalence of those living with the sequelae of noma was placed at 770,000 in 1997 [8].

### Pathogenesis

Infection is multifactorial in nature and not clearly understood. Noma generally arises in the presence of preexisting malnutrition, poor oral hygiene, and an inciting illness, in combination. This triad allows for a polymicrobial infection to take hold. The specific infectious agent and inciting illness are less well-defined. Hypotheses about triggers for the illness include systemic febrile illness (e.g., from measles, varicella, or malaria), neurotrophic viral infection (e.g., herpes), periodontal disease, or other trauma leading to mucosal ulceration. Because of the polymicrobial environment of the oropharynx, it is unclear which infectious agents are the driving forces of the disease. Frequently, identified flora have included *Tremponema vicentii*, other spirochetes or fusiform bacteria, *Staphyloccus aureus*, α-hemolytic *Streptococcus*, and *Pseudomonas* [1], [3], [4]. Lastly, preexisting poor oral hygiene makes superinfection ubiquitous and thus bacteriologic studies potentially misleading [1]. The disease, though considered infectious in origin, is generally believed to be opportunistic rather than communicable [1], [9], [12].

### Clinical Presentation

The clinical presentation of noma includes malodorous breath, excessive salivation, severe dehydration, anemia, and symptoms of both acute and chronic illnesses such as kwashiorkor [1], [9]. Fever, lymphadenopathy, and leukocytosis may reflect the acute infectious process [1], [4], [9]. The World Health Organization classifies acute noma in four stages, ranging from halitosis with gingival bleeding to fulminent necrotizing stomatitis with tissue-sloughing secondary to gangrene [13].

In children, noma can be associated with various stages of growth retardation [3], [7]. Clinicians should be aware that noma is not only found in developing countries and areas of impoverishment. Documented cases of cancrum oris refractory to medical treatment have been observed in patients with immunosupression [14].

### Differential Diagnosis

The differential diagnosis of noma includes leprosy, leishmaniasis, post Kala-azar dermal leishmaniasis (PKDL), oral cancer, acute herpetic gingivostomatitis, syphilitic yaws, mucormycosis, chemical burns, acute necrotizing ulcerative gingivitis (Vincent's disease, or trench mouth), clostridial or streptococcal gangrene, and lethal midline granuloma (also known as Stewart's granuloma or midfacial lymphoma) [1], [15], [16].

### Clinical Course

Noma is devastating clinically and socially. Untreated, 10%–20% of children survive acute noma [8], [17]. Fortunately, treatment with antibiotics and nutrition increases survival of acute childhood noma to greater than 90% [3]. Even with medical treatment, oral disfigurement and frank oral incontinence resulting from the rapidly progressive and consumptive gangrene often requires surgical reconstruction to achieve functionality and, when possible, acceptable cosmesis.

After onset, mucosal ulceration may stay relatively static for a long time before progressing to tender stomatitis and edema [1]. Eventually, a painless necrotic center develops, with outward expansion following a sharp perimeter [3], [4], [9]. Like necrotizing fasciitis, the infection spreads across tissue types and planes [3], [4], [9], [18]. Tissue slough leads to exposure and devitalization of mandibular and maxillary bone, which is rapidly resorbed, leading to staggering facial deformities and oral incontinence [1], [3].

### Treatment

Treatment is aimed at arresting the acute infection and at correcting underlying deficits in nutrition and hydration. Fluid hydration (via nasogastric tube or intravenously in cases of incapacitating oral incontinence), correction of electrolyte abnormalities, nutritional support, vitamin supplementation, and broad spectrum antibiotics are first line [1]. Surgical debridement and plastic reconstruction are frequently necessary after tissue sloughing occurs. Treated or not, death may occur because of underlying illness, systemic inflammatory response, malnutrition secondary to oral incontinence, aspiration pneumonia, or septicemia [19].
